# Interferon-related genetic markers of necroinflammatory activity in chronic hepatitis C

**DOI:** 10.1371/journal.pone.0180927

**Published:** 2017-07-12

**Authors:** Rosario López-Rodríguez, Ángel Hernández-Bartolomé, María Jesús Borque, Yolanda Rodríguez-Muñoz, Samuel Martín-Vílchez, Luisa García-Buey, Leticia González-Moreno, Yolanda Real-Martínez, Paloma Muñoz de Rueda, Javier Salmerón, José Ramón Vidal-Castiñeira, Carlos López-Larrea, Luis Rodrigo, Ricardo Moreno-Otero, Paloma Sanz-Cameno

**Affiliations:** 1 Liver Unit, Gastroenterology Service, Instituto Investigación Sanitaria Princesa, IIS-IP, Madrid, Spain; 2 Molecular Biology Unit, Instituto Investigación Sanitaria Princesa, IIS-IP, Madrid, Spain; 3 CIBERehd, Instituto de Salud Carlos III (ISCIII), Madrid, Spain; 4 Gastroenterology Unit, Hospital Universitario San Cecilio, Granada, Spain; 5 Inmunology Service, Hospital, Universitario Central de Asturias, Oviedo, Spain; 6 Digestive Service, Hospital Universitario Central de Asturias, Oviedo, Spain; University of Navarra School of Medicine and Center for Applied Medical Research (CIMA), SPAIN

## Abstract

**Introduction:**

Chronic hepatitis C (CHC) is a major cause of liver disease worldwide which often leads to progressive liver inflammation, fibrosis, cirrhosis and hepatocellular carcinoma (HCC). CHC displays heterogeneous progression depending on a broad set of factors, some of them intrinsic to each individual such as the patient's genetic profile. This study aims to evaluate the contribution of certain genetic variants of crucial interferon alpha and lambda signaling pathways to the hepatic necroinflammatory activity (NIA) grade of CHC patients.

**Methods:**

NIA was evaluated in 119 CHC patients by METAVIR scale and classified as low (NIA = 0–2, n = 80) or high grade (NIA = 3, n = 39). In a candidate gene approach, 64 SNPs located in 30 different genes related to interferon pathways (*IL-28B*, *IFNAR1-2*, *JAK-STAT* and *OAS1-3*, among others) were genotyped using the Illumina GoldenGate® Genotyping Assay. Statistical association was determined by logistic regression and expressed as OR and 95% CI. Those SNPs significantly associated were further adjusted by other covariates.

**Results:**

Seven SNPs located in *IL-28B* (rs12979860), *JAK1* (rs11576173 and rs1497056), *TYK2* (rs280519), *OAS1* (rs2057778), *SOCS1* (rs33932899) and *RNASEL* (rs3738579) genes were significantly related to severe NIA grade (p<0.05). Regarding to clinical variables, elevated NIA was notably associated with aspartate aminotransferase (AST) serum levels >40 IU/L (p<0.05) but not with other clinical factors. Multivariate logistic regression analysis of these factors reflected that AST (>40 IU/L), *TYK2* rs280519 (G allele) and *RNASEL* rs3738579 (G allele) were factors independently associated with elevated NIA (p<0.05). AST concentration showed a moderate AUC value (AUC = 0.63), similar to TYK2 (rs280519) and *RNASEL* (rs3738579) SNPs (AUC = 0.61, both) in the ROC_AUC analysis. Interestingly, the model including all significant variables reached a considerable predictive value (AUC = 0.74).

**Conclusion:**

The identified genetic variants in interferon signaling pathways may constitute useful prognostic markers of CHC progression. Further validation in larger cohorts of patients is needed.

## Introduction

Hepatitis C virus (HCV) is a major cause of liver-related morbidity and mortality, affecting 170 million people worldwide [1]. Natural history of chronic hepatitis C (CHC) is characterized by a highly variable progression and depending on the extent of liver fibrosis and inflammation CHC can progress to cirrhosis and hepatocellular carcinoma (HCC) [2]. Although new antiviral drugs are highly effective in eradicating HCV infection, there is an important percentage of patients in which the use of these therapies is restricted due to co-morbidities or socio-economic reasons [3]. Moreover, recent studies suggest that virus clearance, especially at advanced stages of disease, does not definitively guarantee healing of liver injury and neither abrogates the risk of liver decompensation or HCC development [4–6]. Therefore, the identification of non-invasive biomarkers that accurately predict the evolution of the disease might notably improve the prevention and clinical management of these patients, especially in the view of unexpected elevated frequency of hepatic and extrahepatic events related to current interferon (IFN)-free therapy. The immune system plays a central role in the therapeutic response and in the appearance of various disorders associated with CHC [7,8], and several genome-wide association studies have revealed the strong association of *IL-28B* rs12979860 polymorphism with the attainment of sustained virological response (SVR) in patients treated with the conventional antiviral therapy (pegylated-interferon and ribavirin) [9–11]. Similarly, other SNPs located in interferon stimulated genes (ISGs) were also independently related to SVR improving the predictive value of *IL-28B* (rs12979860) for combination treatment outcome [12].

On the other hand, the implication of inflammation in the progression of CHC and development of HCC is well established and several inflammatory signaling pathways link inflammation and cancer [13,14]. Therefore, the progression of CHC could be influenced by host genetic variants of innate immunity response genes. Indeed, an important association between the *IL-28B* C/C rs12979860 genotype and higher degree of hepatic inflammation and fibrosis in CHC patients has been described [15–17]. Based on these results, the present study aims to evaluate the contribution of different genetic variants, located in the crucial interferon alpha and lambda signaling pathways, on the hepatic necroinflammatory activity of CHC patients.

## Materials and methods

### Patients and study design

The study population included 119 patients of European descent from Hospital Universitario de La Princesa, Hospital Universitario San Cecilio de Granada and Hospital Universitario Central de Asturias. All patients gave their written informed consent to participate in the genetic analysis before their enrollment. This retrospective cohort study was approved by the local Ethics Committee of Hospital de La Princesa (Madrid, Spain) and good clinical practice guidelines were followed.

Included patients had previous diagnosis of HCV infection, which was confirmed by the presence of detectable HCV RNA in serum. HCV RNA levels and HCV genotype were determined using the COBAS AMPLICOR^®^ assay (Roche Molecular Diagnosis GmbH, Mannheim, Germany) and the reverse-hybridization line probe assay (INNO-LiPAHCV; Innogenetics, Zwijndreht, Belgium), respectively. At Hospital Universitario San Cecilio, viral load was determined the by HCV Ampliprep TaqMan, Roche Molecular System (cutoff <15 IU/mL). Patients showed no evidence of HBV infection, HIV infection, alcoholism, autoimmune, or drug-induced liver disease. Patients’ serum samples were also subjected to routine laboratory tests in the biochemical laboratory of each center using common commercial methods, measuring alanine transaminase (ALT), aspartate aminotransferase (AST) and gamma glutamyl transferase (GGT).

### Necroinflammatory activity assessment

Liver biopsies, obtained for diagnostic purposes, were analyzed histologically according to the METAVIR classification system [18], which scores NIA from 0 to 3: A0, no histologic necroinflammatory activity; A1, minimal activity; A2, moderate activity and A3, severe activity.

### SNP selection and genotyping

DNA was isolated from whole blood samples from Hospital Universitario de La Princesa and Hospital Universitario San Cecilio by using a MagNA Pure DNA isolation kit (Roche Diagnostics, Mannheim, Germany) in accordance with the manufacturer’s instructions and stored at −80°C until assay. Genomic DNA was extracted from peripheral blood samples from Hospital Universitario Central de Asturias with the Magtration-MagaZorb DNA Common Kit-200 N using the Magtration 12GC system (Precision System Science Co., Ltd., Woerrstadt, Germany) and the Maxwell 16 Blood Purification Kit using the Maxwell 16 Instrument (Promega Corporation, Madison, Wisconsin, USA).

A total of 63 SNPs located in 30 interferon signaling pathway and interferon stimulated genes were selected by their tagSNP condition (tagSNPs capture most of the genetic variation in a region) or location in key regulatory regions as described in the HapMap project, Release no. 27, Phase II and III (www.hapmap.org) for the CEU population. The specific location of each SNP (coding/non-coding region and chromosomal position) is detailed in (S1 Table). SNPs with an estimated minor allele frequency of <5%, an estimated r^2^<0.8 between two SNPs in the same gene or with low predicted quality for being genotyped were discarded. Such strategy allows to capture 341 SNPs (r^2^>0.8; S1 Table) from genotyping 63 variants (GoldenGate Genotyping Assay, Illumina Inc., San Diego, CA, USA) at CICbioGUNE (Center for Cooperative Research in Biosciences, Vizcaya, Spain). Complementarily, the *IL-28B* rs12979860 was genotyped by polymerase chain reaction, as described [19].

### Statistical analysis

Hardy–Weinberg equilibrium and allele/genotype frequencies were calculated using SNPStats software [20]. The association between SNPs and NIA grade and the most accurate model of inheritance were determined by logistic regression analysis and expressed as odds ratio, 95% confidence interval and p value.

Clinical variables were expressed as medians and 1st-3rd quantiles or number of patients and percentages, except for age (median and range). Associations between variables and NIA were assessed by Mann-Whitney U-test or Chi-squared test. Two-tailed p values below 0.05 were considered significant (SPSS version 15.0; SPSS Inc., Chicago, IL, USA).

Subsequently, significant clinical variables and SNPs were analyzed by multivariate logistic regression using a step-backward method, in which none of the variables was forced to be included into the model. ROC analyses were performed to discriminate the power of factors independently associated with inflammatory grade (SPSS version 15.0; SPSS Inc., Chicago, IL, USA).

## Results

### Clinical characteristics related to necroinflammatory activity grade

Main clinical characteristics of the 119 patients included in the study are summarized in Table 1. Patients were predominantly men (64%), infected by viral genotype 1 (88%) and with a median age of 45 years at the time of the liver biopsy. According to METAVIR score, 67% of patients presented a moderate grade of necroinflammatory activity (NIA≤2). Patients with severe inflammation score (NIA = 3) showed lower viral load and higher serum levels of AST compared to patients with moderate NIA grade (Table 1). No significant differences were found concerning to the other clinical characteristics.

**Table 1:** Clinical characteristic of the patients included in this study.

	Overall (n = 119)	NIA≤2 (n = 80)	NIA = 3 (n = 39)	p value
Sex (W/M)	36% / 64%	34% / 66%	41% / 59%	ns
VG (1/non1)	88% / 12%	88% / 12%	90% / 10%	ns
Age (years old)	45 (21–67)	46 (21–67)	45 (25–67)	ns
VLx10^-5^ (IU/mL)	11 (5–23)	12 (5–30)	7 (5–13)	0.01
ALT (IU/L)	72 (51–115)	69 (48–102)	82 (60–146)	ns
AST (IU/L)	47 (36–65)	43 (33–58)	58 (41–75)	0.005
GGT (IU/L)	45 (28–83)	41 (27–91)	50 (29–81)	ns
Fibrosis^1^ (FIB≤2/FIB = 3–4)	73% / 27%	77% / 23%	64% / 36%	ns

Data are shown as median and 1st-3rd quantiles except for age (median and range) and sex, viral genotype and fibrosis (percentage of patients).

^1^Only 104 data available.

NIA, necroinflammatory activity; VG, Viral Genotype; VL, Viral Load; W, women; M, men; IU, international units; ALT, alanine transaminase; AST, aspartate aminotransferase; GGT, gamma glutamyl transferase; FIB, fibrosis stage; ns, non significant.

### Genetic variants associated with necroinflammatory activity

Genotype frequencies of each SNP and its association with necroinflammatory activity are detailed in (S2 Table). Of the 64 interferon-related SNPs, five were excluded of the statistical analysis, three monomorphic SNPs (rs11575221, rs11575216 and rs7977692) and two SNPs (rs1061502 and rs17860115), on the basis of their call rate (<80%). SNPs included in the association analysis (n = 59) followed the Hardy–Weinberg equilibrium.

Seven SNPs located in *IL-28B* (rs12979860), *JAK1* (rs11576173 and rs1497056), *OAS1* (rs2057778), *RNASEL* (rs3738579), *TYK2* (rs280519) and *SOCS1* (rs33932899) genes were significantly related to severe NIA grade (Table 2, p<0.05). Interestingly, *IL-28B* T allele, associated previously with poor response to interferon-based therapy, was related to lower NIA score in our group of patients (p = 0.031). In fact, *IL-28B* C/C genotype was more frequent in patients with severe NIA grade (38%) than in patients presenting moderate liver inflammation (25%). *JAK1* rs14907056 G allele showed a higher frequency among patients with severe NIA grade (21% vs. 10% in moderate NIA). Additionally, *JAK1* rs11576173 A/A genotype showed to be more frequent among patients with severe NIA, although this result could be doubtful because of the total number of patients carrying this genotype (n = 5). Similarly, rs2057778 C/C and rs33932899 G/G genotypes located in *OAS1* and *SOCS1*, respectively, showed a slight relation to severe NIA grade; however, only 5 and 3 patients carried the risk genotypes (Table 2). One of the SNPs analyzed in *RNASEL* (rs3738579), an interferon stimulated gene (ISG), displayed an association with liver inflammation, being the G allele related to severe NIA (p = 0.036). *TYK2* rs280519 showed the stronger association with NIA grade: while the A/A genotype was present in near 30% of the patients with moderate NIA, only 8% of the patients with severe liver inflammation carried this genotype (p = 0.009).

**Table 2:** Genetic variants associated with severe necroinflammatory activity in chronic hepatitis C patients.

Locus	SNP	Model	Genotype	NIA≤2	NIA = 3	p value
*IL-28B*	rs12979860	Additive	C/C	20 (25.3%)	15 (38.5%)	0.031
			C/T-T/T	59 (74.7%)	24 (61.5%)	
*JAK1*	rs11576173	Recessive	G/G-A/G	70 (98.6%)	34 (89.5%)	0.034
			A/A	1 (1.4%)	4 (10.5%)	
	rs1497056	Dominant	A/A	60 (81.1%)	21 (60%)	0.021
			A/G-G/G	14 (18.9%)	14 (40%)	
*OAS1*	rs2057778	Recessive	A/A-A/C	74 (97.4%)	34 (87.2%)	0.036
			C/C	2 (2.6%)	5 (12.8%)	
*SOCS1*	rs33932899	Recessive	C/C-G/C	75 (100%)	34 (91.9%)	0.034
			G/G	0 (0%)	3 (8.1%)	
*RNASEL*	rs3738579	Dominant	A/A	33 (43.4%)	9 (23.7%)	0.036
			A/G-G/G	43 (56.6%)	29 (76.3%)	
*TYK2*	rs280519	Recessive	G/G-A/G	50 (71.4%)	34 (91.9%)	0.009
			A/A	20 (28.6%)	3 (8.1%)	

*IL-28B*, interferon lambda 3*; JAK1*, Janus kinase 1*; OAS1*, 2'-5'-oligoadenylate synthetase 1*; RNASEL*, ribonuclease L*; TYK2*, Tyrosine kinase 2*; SOCS1*, Suppressor of cytokine signaling 1. Call rate of each SNP ≥90% (n≥107).

### Multivariate logistic regression and ROC-AUC analyses

Main clinical characteristics and the seven SNPs found in association with NIA grade were included in a multivariate logistic regression analysis (Table 3). Regarding to clinical variables, elevated NIA was notably associated with AST serum levels >40 IU/L (p = 0.01), but not with other clinical factors (such as viral genotype, ALT, GGT or viral load). In addition, *TYK2* rs280519 and *RNASEL* rs3738579 were independent factors strongly associated with elevated NIA (p = 0.02, both).

**Table 3:** Clinical and genetic factors associated with NIA grade in the univariate and multivariate logistic regression analyses.

Factor		Univariate			Multivariate
		OR	CI 95%	p	p
Sex	W	1	-		-
	M	0.73	0.33–1.62	ns	
Viral Genotype	1	1	-		-
	non1	0.8	0.20–2.50	ns	
Age (years old)	≤40	1	-		-
	>40	0.63	0.28–1.41	ns	
Fibrosis stage	FIB≤2	1			
	FIB = 3–4	1.96	0.79–4.89	ns	-
Viral Load (IU/mL)	≤6·10^5^	1	-		-
	>6·10^5^	0.76	0.34–1.74	ns	
ALT (IU/L)	≤40	1	-		-
	>40	0.76	0.23–2.70	ns	
AST (IU/L)	≤40	1	-		0.01
	>40	2.43	1.07–5.86	0.0003	
GGT (IU/L)	≤30	1	-		-
	>30	1.35	0.58–3.31	ns	
*IL-28B*	C/C	1	-		ns
rs12979860	C/T-T/T	0.51	0.27–0.96	0.031	
*JAK1*	A/A	1	-		ns
rs1497056	A/G-G/G	2.86	1.17–6.97	0.021	
*JAK1*	G/G-A/G	1	-		ns
rs11576173	A/A	8.24	0.89–76.53	0.034	
*OAS1*	A/A-A/C	1	-		ns
rs2057778	C/C	5.44	1.01–29.47	0.036	
*RNASEL*	A/A	1	-		0.02
rs3738579	A/G-G/G	2.47	1.03–5.93	0.036	
*TYK2*	G/G-A/G	1	-		0.02
rs280519	A/A	0.22	0.06–0.80	0.009	
*SOCS1*	C/C-G/C	1.00			ns
rs33932899^a^	G/G	NA	NA	0.034	

^a^OR cannot be calculated as the value of one genotypic group is 0.

OR, odds ratio; CI, Confidence interval; W, women; M, men; IU, international units; ALT, alanine transaminase; AST, aspartate aminotransferase; GGT, gamma glutamyl transferase; FIB, fibrosis stage; *IL-28B*, interferon lambda 3*; JAK1*, Janus kinase 1*; OAS1*, 2'-5'-oligoadenylate synthetase 1*; RNASEL*, ribonuclease L*; TYK2*, Tyrosine kinase 2*; SOCS1*, Suppressor of cytokine signaling 1; ns, non significant; NA, Not Applicable.

It must be noted that neither *TYK2* rs280519 nor *RNASEL* rs3738579 SNPs were significantly related to other clinical variables such as viral load, AST, ALT, GGT or fibrosis stage (S3 Table).

**Fig 1:**
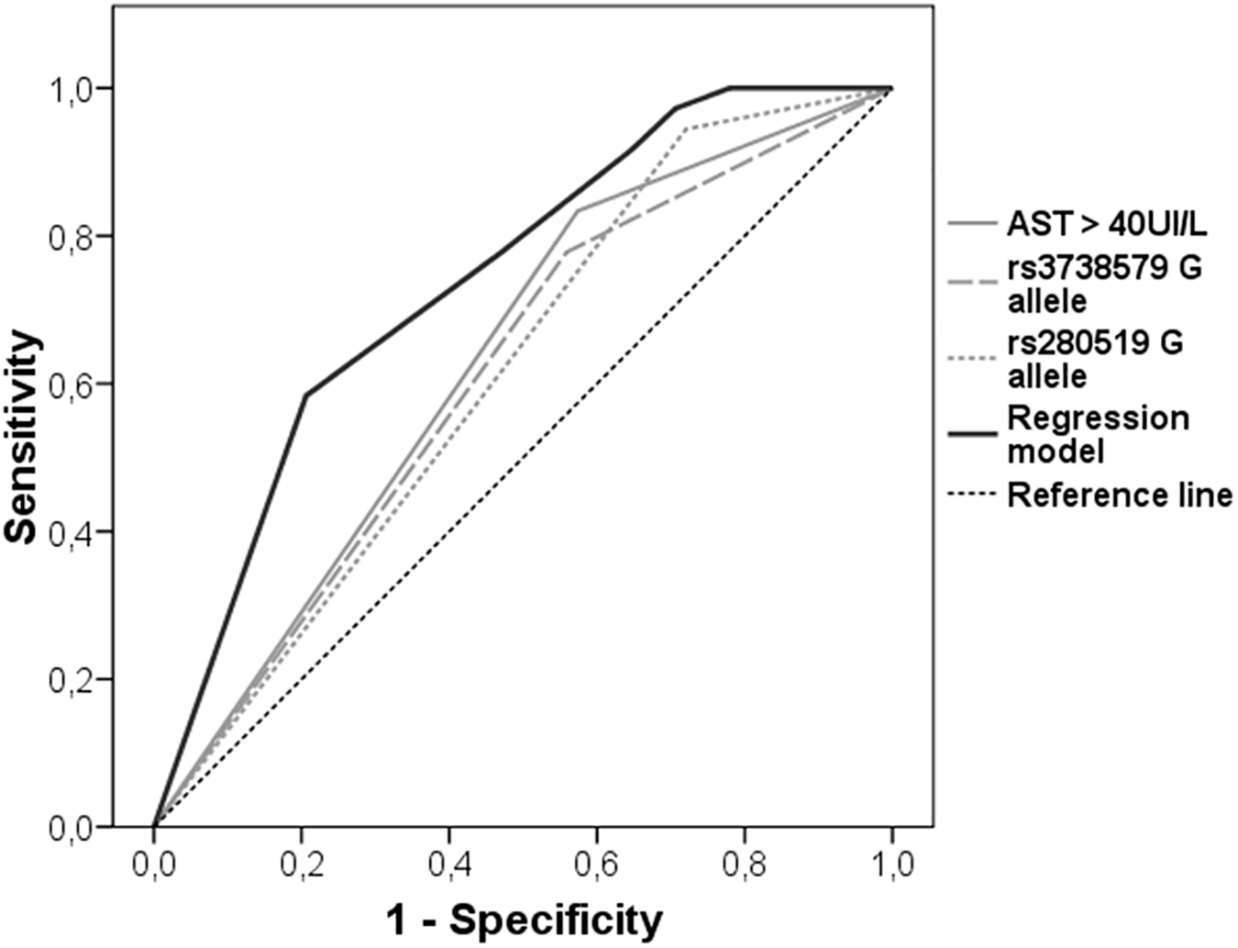
ROC curve analyses of independently associated factors with necroinflammatory activity in chronic hepatitis C patients. AUC of the variables associated with NIA grade: AST serum levels >40IU/mL (AUC = 0.63), risk allele of *RNASEL* rs3738579 and *TYK2* rs280519 (AUC = 0.61, both); and the multivariate regression model including all the above variables (AUC = 0.74).

ROC_AUC analysis of each variable associated with NIA in the multivariate analysis was performed to assess their putative predictive value (Fig 1). AST serum level had the highest AUC value (AUC = 0.63) followed by *TYK2* rs280519 and *RNASEL* rs3738579 (AUC = 0.61, both). Nonetheless, the model including all the above variables showed the best predictive value (AUC = 0.74).

## Discussion

As known, HCV clearance, CHC progression and response to treatment is driven by multiple and complex interactions among host, viral and environmental factors [21]. Innate immunity constitutes the first line of defense against HCV infection and triggers the synthesis of interferons (IFNs), chief antiviral factors able to reprogram cellular status by the induction of multiple genes (IFN-stimulated genes, ISGs) which limit virus replication and the subsequent liver damage [22–24]. Therefore, host genetic polymorphisms in genes involved in IFN signaling may notably influence the onset and progression of the disease as has been demonstrated for *IL-28B* [9–11].

Interestingly, in this study we have inspected multiple SNPs located at numerous IFN related genes finding an independent association of some of them with the NIA of CHC patients. In particular, rs280519 and rs3738579 showed a significant influence in the grade of the disease and displayed a considerable individual predictive performance, which was substantially improved when they were simultaneously considered in addition to AST serum levels (AUC-ROC = 0.74).

The upregulation of many ISGs has been associated with decreased HCV viral RNA levels in the liver of CHC patients as well as in infected hepatocytes [25]. Among them, the OAS/RNaseL pathway constitutes a key antiviral mechanism triggered by IFNs [26]. 2–5OAS proteins are stimulated by IFNs to synthesize 2-5A oligoadenylates that activate RNaseL to cleave dsRNA [27,28]; amazingly, the response of CHC patients to IFN-based treatment is related to RNaseL and it has been described that NS5a can inhibit IFN signaling through its direct binding to 2–5OAS [29]. Hence, the strength of this interaction may depend on different viral strains and it has been suggested as the basis through which IFN-resistant viral genotype 1 evade nucleolytic cleavage [30]. Similarly, the efficiency of OAS/RNaseL pathway might result notably altered by the herein identified genetic variants and could be determinant for the progression of the liver disease. In the present study, we found the G allele of the rs3738579 associated with a higher liver inflammation grade. This SNP located at the 5’ UTR region is in linkage disequilibrium (r^2^>0.8) with other ten SNPs in European descent population (data from the 1000 Genomes Project Phase 3, CEU population [31]). Interestingly, one of them, rs486907, causes an amino acid change (R/Q) at position 462 of RNaseL (S4 Table) which produces a lower RNaseL activity leading to increased cancer risk [32]. This possible effect could explain the higher liver inflammation found in CHC patients carrying the risk allele due to lower efficiency of HCV cleavage. However, further functional studies are needed to confirm these results. The other SNPs in linkage disequilibrium with rs3738579, located upstream and downstream *RNaseL*, have not been related to functional or transcriptional changes to date (S4 Table).

Tyk2 signaling is shared by both type I and III IFNs and its altered expression as well as some Tyk2 genetic variants have been implicated in the pathogenesis of numerous autoimmune diseases such as rheumatoid arthritis, systemic lupus erythematosus, multiple sclerosis, diabetes, ulcerative colitis and Crohn's disease [33]. Furthermore, lower Tyk2 expression levels were observed in HCV subgenomic replicon cell-lines unable to respond to IFNλ [34]. On the other hand, HCV Core reduced the phosphorylation levels of Tyk2 and Jak1, limiting the response to IFN-α [35]. Interestingly, rs280519 G allele was related to severe NIA grade in CHC patients in this study. Although this SNP does not cause an amino acid variant, its location in the splice site of the intronic region of *TYK2* makes this SNP of particular interest for transcriptional regulation. Four SNPs were found in linkage disequilibrium with rs280519 in the last data released by The 1000 Genomes Project (Phase 3 [31]), all of them located at intron regions except for rs280497 whose G allele seems to affect a transcriptional binding site at promoter flaking region (data from Ensembl [36,37]). In addition, *TYK2* is located at Chromosome 19 and surrounded by genes involved in key immune processes such as *ICAM* (S1 Fig from SNAP [38]). Those findings highlight the importance of developing an in depth functional study focused on elucidating the prognostic potential of rs280519 or the linked SNPs for the progression of CHC and other inflammatory pathologies.

Further validation of the above described genetic signature may entail a great interest for accurate prediction of CHC patients with higher risk of CHC progression, with special attention to the unexpected elevated occurrence/recurrence of hepatic and extrahepatic manifestations related to current IFN-free based therapies.

## Supporting information

S1 Table Genotyped SNPs, location and SNPs in linkage disequilibrium with an r^2^>0.8 in the CEU population. (DOCX)

S2 Table Genotypic distribution of the analyzed SPNs in the overall population of CHC patients and stratified by necroinflammatory activity grade. (DOCX)

S3 Table Distribution of main clinical variables among *TYK2* rs280519 and *RNASEL* rs3738579 genotypes. (DOCX)

S4 Table Variants linked to *TYK2* rs280519 and *RNASEL* rs3738579. (DOCX)

S1 Fig TYK2 rs280519 linkage disequilibrium plot. Linkage disequilibrium plot of rs280519 was generated by using the SNP Annotation and Proxy Search tool (SNAP, version 2.2, Broad Institute [38]). Plot was generated under the following conditions: 1000 Genomes Pilot 1 dataset, CEU population panel, r^2^ threshold = 0.8 and distance limit 500kb.
(PDF)
