# Supplementary material for: Interferon-related genetic markers of necroinflammatory activity in chronic hepatitis C
Source: PLoS One. 2017 Jul 12;12(7):e0180927. doi: 10.1371/journal.pone.0180927 (PMC5507534; doi:10.1371/journal.pone.0180927)
Supplement: S1 Table — (DOCX) [file pone.0180927.s001.docx]

**S1 Table. Genotyped SNPs , location and SNPs in Linkage disequilibrium with an r^2^>0.8 in the CEU population**

| tagSNP | MAF | Chr | Gene symbol | Location | Coding status | Amino acid  change | SNPs  in LD | Distance (bp) | r^2^ | D’ |
| --- | --- | --- | --- | --- | --- | --- | --- | --- | --- | --- |
| rs10207436 | G:0.2624 | 2 | PRKRA | flanking_3UTR |  |  | rs10207436 | 0 | 1.000 | 1.000 |
|  |  |  |  |  |  |  | rs2114582 | 6777 | 0.977 | 1.000 |
|  |  |  |  |  |  |  | rs2288322 | 28506 | 0.977 | 1.000 |
| rs10208033 | C:0.4374 | 2 | STAT1 | flanking_5UTR |  |  | rs10208033 | 0 | 1.000 | 1.000 |
|  |  |  |  |  |  |  | rs6751855 | 5354 | 0.872 | 1.000 |
| rs10415576 | C:0.3787 | 19 | IRF3 | intron |  |  | rs10415576 | 0 | 1.000 | 1.000 |
| rs1048260 | C:0.2942 | 1 | RNASEL | 3UTR |  |  | rs1048260 | 0 | 1.000 | 1.000 |
| rs1049493 | C: 0.442 | 15 | PIAS1 | flanking_3UTR |  |  | rs1049493 | 0 | 1.000 | 1.000 |
|  |  |  |  |  |  |  | rs11071986 | 11141 | 0.983 | 1.000 |
|  |  |  |  |  |  |  | rs8024471 | 41963 | 0.983 | 1.000 |
|  |  |  |  |  |  |  | rs2169215 | 57969 | 0.983 | 1.000 |
|  |  |  |  |  |  |  | rs11629609 | 59644 | 0.983 | 1.000 |
|  |  |  |  |  |  |  | rs8038170 | 62241 | 0.983 | 1.000 |
|  |  |  |  |  |  |  | rs1554223 | 64716 | 0.983 | 1.000 |
|  |  |  |  |  |  |  | rs8030302 | 39402 | 0.966 | 1.000 |
|  |  |  |  |  |  |  | rs12912276 | 695 | 0.949 | 1.000 |
|  |  |  |  |  |  |  | rs11635988 | 11375 | 0.949 | 1.000 |
|  |  |  |  |  |  |  | rs11071987 | 14867 | 0.949 | 1.000 |
|  |  |  |  |  |  |  | rs11071991 | 17165 | 0.949 | 1.000 |
|  |  |  |  |  |  |  | rs10431808 | 26385 | 0.949 | 1.000 |
|  |  |  |  |  |  |  | rs8035890 | 43785 | 0.949 | 0.983 |
|  |  |  |  |  |  |  | rs12904794 | 45896 | 0.949 | 1.000 |
|  |  |  |  |  |  |  | rs7166850 | 46597 | 0.949 | 1.000 |
|  |  |  |  |  |  |  | rs4777022 | 40859 | 0.933 | 1.000 |
|  |  |  |  |  |  |  | rs7167918 | 48747 | 0.933 | 1.000 |
|  |  |  |  |  |  |  | rs7165825 | 49028 | 0.933 | 1.000 |
|  |  |  |  |  |  |  | rs4777023 | 26935 | 0.932 | 0.982 |
|  |  |  |  |  |  |  | rs12905763 | 31136 | 0.932 | 0.982 |
|  |  |  |  |  |  |  | rs8025947 | 29883 | 0.917 | 1.000 |
|  |  |  |  |  |  |  | rs11856619 | 76525 | 0.917 | 1.000 |
|  |  |  |  |  |  |  | rs7402649 | 44312 | 0.916 | 0.982 |
|  |  |  |  |  |  |  | rs8028241 | 18434 | 0.915 | 0.965 |
|  |  |  |  |  |  |  | rs8038236 | 55622 | 0.901 | 1.000 |
|  |  |  |  |  |  |  | rs12323992 | 63415 | 0.901 | 1.000 |
|  |  |  |  |  |  |  | rs7162430 | 73600 | 0.900 | 0.982 |
|  |  |  |  |  |  |  | rs8031079 | 79305 | 0.900 | 0.982 |
|  |  |  |  |  |  |  | rs6494711 | 106860 | 0.900 | 0.982 |
|  |  |  |  |  |  |  | rs11636657 | 127443 | 0.853 | 0.964 |
|  |  |  |  |  |  |  | rs11635759 | 140494 | 0.852 | 0.947 |
|  |  |  |  |  |  |  | rs12905500 | 127311 | 0.820 | 0.929 |
| rs10502878 | T:0.1948 | 18 | PIAS2 | flanking_3UTR |  |  | rs10502878 | 0 | 1.000 | 1.000 |
|  |  |  |  |  |  |  | rs17708203 | 29788 | 1.000 | 1.000 |
|  |  |  |  |  |  |  | rs12457793 | 173586 | 0.971 | 1.000 |
| rs1051042 | G: 0.345 | 12 | OAS1 | coding |  |  | rs1051042 | 0 | 1.000 | 1.000 |
| rs1061502 | C: 0.267 | 11 | IRF7 | coding | NONSYN | K179E | rs1061502 | 0 | 1.000 | 1.000 |
| rs10735079 | G: 0.364 | 12 | OAS3 | intron |  |  | rs10735079 | 0 | 1.000 | 1.000 |
|  |  |  |  |  |  |  | rs1156361 | 4025 | 0.965 | 1.000 |
|  |  |  |  |  |  |  | rs4767040 | 5991 | 0.965 | 1.000 |
|  |  |  |  |  |  |  | rs2269899 | 1948 | 0.964 | 1.000 |
|  |  |  |  |  |  |  | rs10774679 | 5260 | 0.948 | 1.000 |
|  |  |  |  |  |  |  | rs1859331 | 3677 | 0.947 | 0.982 |
|  |  |  |  |  |  |  | rs1859333 | 9042 | 0.947 | 0.982 |
|  |  |  |  |  |  |  | rs7132404 | 11403 | 0.947 | 0.982 |
|  |  |  |  |  |  |  | rs1859336 | 12699 | 0.947 | 0.982 |
|  |  |  |  |  |  |  | rs7315441 | 14321 | 0.947 | 0.982 |
|  |  |  |  |  |  |  | rs4766674 | 14807 | 0.947 | 0.982 |
|  |  |  |  |  |  |  | rs6489868 | 16316 | 0.947 | 0.982 |
|  |  |  |  |  |  |  | rs10774676 | 17601 | 0.947 | 0.982 |
|  |  |  |  |  |  |  | rs7966314 | 7469 | 0.930 | 0.982 |
|  |  |  |  |  |  |  | rs10850098 | 17950 | 0.929 | 0.964 |
|  |  |  |  |  |  |  | rs7134391 | 13317 | 0.913 | 0.982 |
|  |  |  |  |  |  |  | rs4766676 | 14427 | 0.878 | 0.945 |
|  |  |  |  |  |  |  | rs2285932 | 6942 | 0.845 | 0.980 |
|  |  |  |  |  |  |  | rs1557866 | 25173 | 0.845 | 0.980 |
|  |  |  |  |  |  |  | rs10744791 | 30308 | 0.845 | 0.980 |
|  |  |  |  |  |  |  | rs10850094 | 19445 | 0.828 | 0.961 |
|  |  |  |  |  |  |  | rs4767030 | 20431 | 0.828 | 0.961 |
|  |  |  |  |  |  |  | rs4767024 | 21217 | 0.828 | 0.961 |
|  |  |  |  |  |  |  | rs3177979 | 22799 | 0.828 | 0.961 |
|  |  |  |  |  |  |  | rs1293764 | 45671 | 0.828 | 0.961 |
| rs11575216 | C: 0.002 | 12 | APOF | coding | NONSYN | A178G | rs11575216 | 0 | 1.000 | 1.000 |
| rs11575221 | G: 0.002 | 12 | APOF | flanking_3UTR |  |  | rs11575221 | 0 | 1.000 | 1.000 |
|  |  |  |  |  |  |  | rs4301822 | 921 | 1.000 | 1.000 |
|  |  |  |  |  |  |  | rs11171818 | 9128 | 1.000 | 1.000 |
|  |  |  |  |  |  |  | rs11171819 | 9307 | 1.000 | 1.000 |
|  |  |  |  |  |  |  | rs7979812 | 27307 | 1.000 | 1.000 |
|  |  |  |  |  |  |  | rs2629445 | 64289 | 1.000 | 1.000 |
|  |  |  |  |  |  |  | rs17118461 | 66332 | 1.000 | 1.000 |
|  |  |  |  |  |  |  | rs6581093 | 70651 | 1.000 | 1.000 |
|  |  |  |  |  |  |  | rs7962107 | 149648 | 1.000 | 1.000 |
|  |  |  |  |  |  |  | rs773658 | 150303 | 1.000 | 1.000 |
|  |  |  |  |  |  |  | rs7489249 | 161106 | 1.000 | 1.000 |
|  |  |  |  |  |  |  | rs2695783 | 165684 | 1.000 | 1.000 |
|  |  |  |  |  |  |  | rs11834728 | 192896 | 1.000 | 1.000 |
|  |  |  |  |  |  |  | rs11171753 | 224146 | 1.000 | 1.000 |
|  |  |  |  |  |  |  | rs1689518 | 296516 | 1.000 | 1.000 |
|  |  |  |  |  |  |  | rs705697 | 446262 | 1.000 | 1.000 |
|  |  |  |  |  |  |  | rs1689509 | 450866 | 1.000 | 1.000 |
| rs11576173 | A: 0.282 | 1 | JAK1 | flanking_5UTR |  |  | rs11576173 | 0 | 1.000 | 1.000 |
|  |  |  |  |  |  |  | rs12743599 | 357 | 1.000 | 1.000 |
| rs12135247 | C: 0.303 | 1 | RNASEL | 3UTR |  |  | rs12135247 | 0 | 1.000 | 1.000 |
|  |  |  |  |  |  |  | rs11072 | 606 | 0.979 | 1.000 |
|  |  |  |  |  |  |  | rs11807829 | 878 | 0.902 | 1.000 |
| rs12436555 | A: 0.131 | 14 | IRF9 | 24165616 |  |  | rs12436555 | 0 | 1.000 | 1.000 |
| rs12805435 | C: 0.267 | 11 | KIAA1542 | flanking_3UTR |  |  | rs12805435 | 0 | 1.000 | 1.000 |
|  |  |  |  |  |  |  | rs10902178 | 488 | 1.000 | 1.000 |
|  |  |  |  |  |  |  | rs11246213 | 612 | 1.000 | 1.000 |
|  |  |  |  |  |  |  | rs11246217 | 11410 | 0.976 | 1.000 |
|  |  |  |  |  |  |  | rs7936397 | 34821 | 0.952 | 1.000 |
| rs12819210 | T: 0.167 | 12 | OASL | coding | SYNON | S503S | rs12819210 | 0 | 1.000 | 1.000 |
| rs12819767 | T: 0.027 | 12 | OAS3 | coding | NONSYN | R65W | rs12819767 | 0 | 1.000 | 1.000 |
| rs1293748 | T: 0.308 | 12 | OAS2 | intron |  |  | rs1293748 | 0 | 1.000 | 1.000 |
| rs1293764 | T: 0.324 | 12 | OAS2 | 3UTR |  |  | rs1293764 | 0 | 1.000 | 1.000 |
|  |  |  |  |  |  |  | rs10744791 | 15363 | 0.982 | 1.000 |
|  |  |  |  |  |  |  | rs1557866 | 20498 | 0.982 | 1.000 |
|  |  |  |  |  |  |  | rs2285932 | 38729 | 0.982 | 1.000 |
|  |  |  |  |  |  |  | rs2269899 | 43723 | 0.860 | 0.962 |
|  |  |  |  |  |  |  | rs10735079 | 45671 | 0.828 | 0.961 |
| rs1293767 | C: 0.325 | 12 | OAS2 | coding | NONSYN | S163R | rs1293767 | 0 | 1.000 | 1.000 |
| rs12979860 | T: 0.309 | 19 | IFNL4 | 101180976 |  |  | rs12979860 | 0 | 1.000 | 1.000 |
| rs1497056 | C: 0.179 | 1 | JAK1 | flanking_5UTR |  |  | rs1497056 | 0 | 1.000 | 1.000 |
|  |  |  |  |  |  |  | rs12745769 | 36318 | 0.860 | 1.000 |
|  |  |  |  |  |  |  | rs10889503 | 40016 | 0.860 | 1.000 |
|  |  |  |  |  |  |  | rs11208538 | 40562 | 0.860 | 1.000 |
| rs17860115 | A: 0.331 | 21 | IFNAR2 | 5UTR |  |  | rs17860115 | 0 | 1.000 | 1.000 |
| rs17860241 | G: 0.325 | 21 | IFNAR2 | 33258828 |  |  | rs17860241 | 0 | 1.000 | 1.000 |
| rs2030171 | A: 0.359 | 2 | STAT1 | intron |  |  | rs2030171 | 0 | 1.000 | 1.000 |
|  |  |  |  |  |  |  | rs10173099 | 1831 | 1.000 | 1.000 |
|  |  |  |  |  |  |  | rs13029247 | 2505 | 0.854 | 0.979 |
| rs2031564 | A: 0.129 | 14 | EIF2S1 | intron |  |  | rs2031564 | 0 | 1.000 | 1.000 |
|  |  |  |  |  |  |  | rs2319777 | 17483 | 0.962 | 1.000 |
|  |  |  |  |  |  |  | rs6573760 | 150141 | 0.962 | 1.000 |
|  |  |  |  |  |  |  | rs8004488 | 160114 | 0.962 | 1.000 |
|  |  |  |  |  |  |  | rs2281676 | 189061 | 0.926 | 1.000 |
|  |  |  |  |  |  |  | rs7151898 | 200922 | 0.926 | 1.000 |
|  |  |  |  |  |  |  | rs7148425 | 204700 | 0.926 | 1.000 |
|  |  |  |  |  |  |  | rs7148044 | 204946 | 0.926 | 1.000 |
|  |  |  |  |  |  |  | rs8010261 | 219399 | 0.926 | 1.000 |
|  |  |  |  |  |  |  | rs1918483 | 219893 | 0.926 | 1.000 |
|  |  |  |  |  |  |  | rs6573752 | 237724 | 0.926 | 1.000 |
|  |  |  |  |  |  |  | rs8012776 | 239638 | 0.926 | 1.000 |
|  |  |  |  |  |  |  | rs2039055 | 246723 | 0.926 | 1.000 |
|  |  |  |  |  |  |  | rs8009087 | 263306 | 0.926 | 1.000 |
|  |  |  |  |  |  |  | rs7157418 | 264701 | 0.926 | 1.000 |
|  |  |  |  |  |  |  | rs7158228 | 264793 | 0.926 | 1.000 |
|  |  |  |  |  |  |  | rs10138952 | 291497 | 0.926 | 1.000 |
|  |  |  |  |  |  |  | rs8003929 | 294243 | 0.926 | 1.000 |
|  |  |  |  |  |  |  | rs9323491 | 300281 | 0.926 | 1.000 |
|  |  |  |  |  |  |  | rs2144061 | 300954 | 0.926 | 1.000 |
|  |  |  |  |  |  |  | rs9323490 | 301179 | 0.926 | 1.000 |
|  |  |  |  |  |  |  | rs7141519 | 302991 | 0.926 | 1.000 |
|  |  |  |  |  |  |  | rs7161664 | 303029 | 0.926 | 1.000 |
|  |  |  |  |  |  |  | rs8021098 | 321309 | 0.926 | 1.000 |
|  |  |  |  |  |  |  | rs7149905 | 327800 | 0.926 | 1.000 |
|  |  |  |  |  |  |  | rs1546940 | 333738 | 0.926 | 1.000 |
|  |  |  |  |  |  |  | rs10142059 | 344887 | 0.926 | 1.000 |
|  |  |  |  |  |  |  | rs1955606 | 349869 | 0.926 | 1.000 |
|  |  |  |  |  |  |  | rs8018750 | 391949 | 0.926 | 1.000 |
|  |  |  |  |  |  |  | rs1950281 | 426217 | 0.926 | 1.000 |
|  |  |  |  |  |  |  | rs8022657 | 437017 | 0.926 | 1.000 |
|  |  |  |  |  |  |  | rs723432 | 448501 | 0.926 | 1.000 |
|  |  |  |  |  |  |  | rs728647 | 449461 | 0.926 | 1.000 |
|  |  |  |  |  |  |  | rs7156655 | 471455 | 0.926 | 1.000 |
|  |  |  |  |  |  |  | rs1950689 | 488838 | 0.926 | 1.000 |
|  |  |  |  |  |  |  | rs11158653 | 168621 | 0.893 | 1.000 |
|  |  |  |  |  |  |  | rs28817965 | 408644 | 0.893 | 1.000 |
|  |  |  |  |  |  |  | rs10138850 | 220748 | 0.887 | 0.960 |
|  |  |  |  |  |  |  | rs1950282 | 339887 | 0.887 | 0.960 |
|  |  |  |  |  |  |  | rs17104114 | 160006 | 0.884 | 0.959 |
|  |  |  |  |  |  |  | rs10135117 | 428125 | 0.884 | 0.959 |
|  |  |  |  |  |  |  | rs10150145 | 300488 | 0.862 | 1.000 |
|  |  |  |  |  |  |  | rs8004148 | 141086 | 0.816 | 0.921 |
| rs2032215 | A: 0.432 | 18 | PIAS2 | 46859048 |  |  | rs2032215 | 0 | 1.000 | 1.000 |
|  |  |  |  |  |  |  | rs658756 | 7179 | 0.982 | 1.000 |
|  |  |  |  |  |  |  | rs626217 | 9829 | 0.982 | 1.000 |
|  |  |  |  |  |  |  | rs2156050 | 16862 | 0.982 | 1.000 |
|  |  |  |  |  |  |  | rs7240239 | 21026 | 0.982 | 1.000 |
|  |  |  |  |  |  |  | rs553456 | 26136 | 0.982 | 1.000 |
|  |  |  |  |  |  |  | rs642897 | 42094 | 0.982 | 1.000 |
|  |  |  |  |  |  |  | rs509647 | 55336 | 0.982 | 1.000 |
|  |  |  |  |  |  |  | rs6507709 | 14127 | 0.965 | 0.982 |
|  |  |  |  |  |  |  | rs649076 | 24397 | 0.965 | 1.000 |
|  |  |  |  |  |  |  | rs10853545 | 36209 | 0.965 | 0.982 |
|  |  |  |  |  |  |  | rs4890341 | 28357 | 0.947 | 0.982 |
|  |  |  |  |  |  |  | rs577200 | 3391 | 0.931 | 1.000 |
|  |  |  |  |  |  |  | rs1539878 | 83993 | 0.914 | 0.982 |
|  |  |  |  |  |  |  | rs2187092 | 87787 | 0.914 | 0.982 |
|  |  |  |  |  |  |  | rs10853547 | 91537 | 0.898 | 0.982 |
|  |  |  |  |  |  |  | rs7244778 | 102904 | 0.898 | 0.982 |
|  |  |  |  |  |  |  | rs2010834 | 121864 | 0.898 | 0.982 |
|  |  |  |  |  |  |  | rs9304337 | 54083 | 0.897 | 0.981 |
| rs2057778 | G: 0.344 | 12 | OAS1 | intron |  |  | rs2057778 | 0 | 1.000 | 1.000 |
|  |  |  |  |  |  |  | rs3177979 | 6413 | 0.963 | 0.981 |
|  |  |  |  |  |  |  | rs4767024 | 7995 | 0.963 | 0.981 |
|  |  |  |  |  |  |  | rs4767030 | 8781 | 0.963 | 0.981 |
|  |  |  |  |  |  |  | rs10850094 | 9767 | 0.963 | 0.981 |
|  |  |  |  |  |  |  | rs10774671 | 6397 | 0.928 | 0.981 |
|  |  |  |  |  |  |  | rs2285934 | 724 | 0.927 | 0.963 |
|  |  |  |  |  |  |  | rs10774676 | 11611 | 0.846 | 0.981 |
|  |  |  |  |  |  |  | rs6489868 | 12896 | 0.846 | 0.981 |
|  |  |  |  |  |  |  | rs4766674 | 14405 | 0.846 | 0.981 |
|  |  |  |  |  |  |  | rs4766676 | 14785 | 0.846 | 0.981 |
|  |  |  |  |  |  |  | rs7315441 | 14891 | 0.846 | 0.981 |
|  |  |  |  |  |  |  | rs1859336 | 16513 | 0.846 | 0.981 |
|  |  |  |  |  |  |  | rs7132404 | 17809 | 0.846 | 0.981 |
|  |  |  |  |  |  |  | rs1859333 | 20170 | 0.846 | 0.981 |
|  |  |  |  |  |  |  | rs4767040 | 23221 | 0.831 | 0.980 |
|  |  |  |  |  |  |  | rs1156361 | 25187 | 0.831 | 0.980 |
|  |  |  |  |  |  |  | rs10850098 | 11262 | 0.828 | 0.961 |
|  |  |  |  |  |  |  | rs7134391 | 15895 | 0.816 | 0.980 |
|  |  |  |  |  |  |  | rs10774679 | 23952 | 0.816 | 0.980 |
| rs2059691 | A: 0.300 | 2 | PRKRA | intron |  |  | rs2059691 | 0 | 1.000 | 1.000 |
|  |  |  |  |  |  |  | rs2249737 | 61801 | 0.939 | 0.979 |
| rs2070845 | G: 0.249 | 10 | IFIT2 | coding | NONSYN | K121R | rs2070845 | 0 | 1.000 | 1.000 |
| rs2072593 | C: 0.222 | 12 | SOCS2 | flanking_3UTR |  |  | rs2072593 | 0 | 1.000 | 1.000 |
| rs2285933 | G: 0.256 | 12 | OAS3 | coding | NONSYN | S381R | rs2285933 | 0 | 1.000 | 1.000 |
| rs2304206 | A: 0.259 | 19 | BCL2L12 | 5UTR |  |  | rs2304206 | 0 | 1.000 | 1.000 |
|  |  |  |  |  |  |  | rs2304204 | 149 | 1.000 | 1.000 |
|  |  |  |  |  |  |  | rs7259683 | 15539 | 1.000 | 1.000 |
| rs2384075 | A: 0.271 | 12 | OAS2 | intron |  |  | rs2384075 | 0 | 1.000 | 1.000 |
|  |  |  |  |  |  |  | rs12422608 | 7805 | 0.919 | 0.959 |
|  |  |  |  |  |  |  | rs12425514 | 8884 | 0.880 | 0.938 |
|  |  |  |  |  |  |  | rs7138267 | 6672 | 0.860 | 0.957 |
|  |  |  |  |  |  |  | rs7023 | 5678 | 0.822 | 0.956 |
|  |  |  |  |  |  |  | rs739903 | 7779 | 0.822 | 0.956 |
|  |  |  |  |  |  |  | rs757404 | 9461 | 0.822 | 0.956 |
|  |  |  |  |  |  |  | rs2240187 | 12658 | 0.822 | 0.956 |
|  |  |  |  |  |  |  | rs2240188 | 12969 | 0.822 | 0.956 |
|  |  |  |  |  |  |  | rs2240189 | 13156 | 0.822 | 0.956 |
|  |  |  |  |  |  |  | rs2072135 | 17474 | 0.803 | 0.955 |
|  |  |  |  |  |  |  | rs7965570 | 30029 | 0.803 | 0.955 |
| rs243330 | T: 0.466 | 16 | SOCS1 | flanking_5UTR |  |  | rs243330 | 0 | 1.000 | 1.000 |
|  |  |  |  |  |  |  | rs11640138 | 11738 | 0.983 | 1.000 |
|  |  |  |  |  |  |  | rs243327 | 2313 | 0.950 | 1.000 |
|  |  |  |  |  |  |  | rs243324 | 3979 | 0.950 | 1.000 |
|  |  |  |  |  |  |  | rs415595 | 12701 | 0.949 | 0.983 |
|  |  |  |  |  |  |  | rs416603 | 13088 | 0.949 | 0.983 |
| rs280519 | A: 0.495 | 19 | TYK2 | intron |  |  | rs280519 | 0 | 1.000 | 1.000 |
| rs2834202 | G: 0.283 | 21 | IFNAR1 | 3UTR |  |  | rs2834202 | 0 | 1.000 | 1.000 |
|  |  |  |  |  |  |  | rs2856973 | 4827 | 1.000 | 1.000 |
|  |  |  |  |  |  |  | rs914142 | 5147 | 1.000 | 1.000 |
|  |  |  |  |  |  |  | rs11700514 | 11219 | 1.000 | 1.000 |
|  |  |  |  |  |  |  | rs2834206 | 11301 | 1.000 | 1.000 |
|  |  |  |  |  |  |  | rs2834183 | 49183 | 0.915 | 0.978 |
|  |  |  |  |  |  |  | rs2834178 | 53563 | 0.915 | 0.978 |
| rs303217 | T: 0.476 | 10 | IFIT1 | intron |  |  | rs303217 | 0 | 1.000 | 1.000 |
|  |  |  |  |  |  |  | rs304494 | 10170 | 1.000 | 1.000 |
|  |  |  |  |  |  |  | rs304504 | 15873 | 1.000 | 1.000 |
|  |  |  |  |  |  |  | rs304478 | 2777 | 0.983 | 1.000 |
|  |  |  |  |  |  |  | rs627524 | 36909 | 0.933 | 0.983 |
|  |  |  |  |  |  |  | rs10749606 | 56631 | 0.933 | 0.983 |
|  |  |  |  |  |  |  | rs10159774 | 50279 | 0.916 | 0.966 |
|  |  |  |  |  |  |  | rs215493 | 10471 | 0.872 | 1.000 |
|  |  |  |  |  |  |  | rs303172 | 26130 | 0.855 | 0.982 |
|  |  |  |  |  |  |  | rs304448 | 24301 | 0.839 | 0.964 |
|  |  |  |  |  |  |  | rs1888923 | 82749 | 0.822 | 0.946 |
|  |  |  |  |  |  |  | rs4934471 | 82029 | 0.821 | 0.930 |
| rs304478 | G: 0.478 | 10 | IFIT1 | flanking_5UTR |  |  | rs304478 | 0 | 1.000 | 1.000 |
|  |  |  |  |  |  |  | rs303217 | 2777 | 0.983 | 1.000 |
|  |  |  |  |  |  |  | rs304494 | 7393 | 0.983 | 1.000 |
|  |  |  |  |  |  |  | rs304504 | 13096 | 0.983 | 1.000 |
|  |  |  |  |  |  |  | rs627524 | 34132 | 0.917 | 0.982 |
|  |  |  |  |  |  |  | rs10749606 | 53854 | 0.917 | 0.982 |
|  |  |  |  |  |  |  | rs10159774 | 47502 | 0.900 | 0.965 |
|  |  |  |  |  |  |  | rs215493 | 13248 | 0.857 | 1.000 |
|  |  |  |  |  |  |  | rs304448 | 27078 | 0.855 | 0.982 |
|  |  |  |  |  |  |  | rs303172 | 28907 | 0.840 | 0.982 |
|  |  |  |  |  |  |  | rs4934471 | 79252 | 0.837 | 0.947 |
|  |  |  |  |  |  |  | rs1888923 | 79972 | 0.808 | 0.946 |
| rs310209 | A: 0.263 | 1 | JAK1 | intron |  |  | rs310209 | 0 | 1.000 | 1.000 |
|  |  |  |  |  |  |  | rs310231 | 4799 | 1.000 | 1.000 |
|  |  |  |  |  |  |  | rs310230 | 4614 | 0.977 | 1.000 |
|  |  |  |  |  |  |  | rs310236 | 11759 | 0.816 | 1.000 |
| rs310216 | A: 0.251 | 1 | JAK1 | intron |  |  | rs310216 | 0 | 1.000 | 1.000 |
|  |  |  |  |  |  |  | rs310219 | 1377 | 1.000 | 1.000 |
|  |  |  |  |  |  |  | rs310225 | 8008 | 1.000 | 1.000 |
|  |  |  |  |  |  |  | rs2780902 | 12425 | 1.000 | 1.000 |
|  |  |  |  |  |  |  | rs2780896 | 5558 | 0.976 | 1.000 |
|  |  |  |  |  |  |  | rs2780820 | 7673 | 0.976 | 1.000 |
|  |  |  |  |  |  |  | rs2780895 | 7275 | 0.952 | 0.976 |
|  |  |  |  |  |  |  | rs310241 | 13337 | 0.952 | 1.000 |
| rs310245 | T: 0.368 | 1 | JAK1 | intron |  |  | rs310245 | 0 | 1.000 | 1.000 |
|  |  |  |  |  |  |  | rs310247 | 1227 | 0.836 | 1.000 |
|  |  |  |  |  |  |  | rs1048007 | 8942 | 0.813 | 0.943 |
| rs3153 | A: 0.296 | 21 | IFNAR2 | 33237200 |  |  | rs3153 | 0 | 1.000 | 1.000 |
| rs3213545 | A: 0.307 | 12 | OASL | coding | SYNON | L136L | rs3213545 | 0 | 1.000 | 1.000 |
|  |  |  |  |  |  |  | rs1182933 | 16715 | 0.882 | 0.939 |
|  |  |  |  |  |  |  | rs2464196 | 35910 | 0.863 | 0.938 |
|  |  |  |  |  |  |  | rs1169300 | 40112 | 0.863 | 0.938 |
|  |  |  |  |  |  |  | rs1169314 | 28221 | 0.825 | 0.937 |
| rs33932899 | G: 0.269 | 16 | SOCS1 | flanking_3UTR |  |  | rs33932899 | 0 | 1.000 | 1.000 |
| rs3738579 | G: 0.360 | 1 | RNASEL | 5UTR |  |  | rs3738579 | 0 | 1.000 | 1.000 |
|  |  |  |  |  |  |  | rs579006 | 6700 | 0.964 | 1.000 |
|  |  |  |  |  |  |  | rs486907 | 1479 | 0.947 | 1.000 |
|  |  |  |  |  |  |  | rs12041623 | 5597 | 0.878 | 0.963 |
|  |  |  |  |  |  |  | rs4652733 | 11404 | 0.878 | 0.963 |
|  |  |  |  |  |  |  | rs3795485 | 11703 | 0.878 | 0.963 |
|  |  |  |  |  |  |  | rs10489966 | 19340 | 0.878 | 0.963 |
| rs3740027 | T: 0.244 | 10 | IFIT2 | flanking_3UTR |  |  | rs3740027 | 0 | 1.000 | 1.000 |
|  |  |  |  |  |  |  | rs11203063 | 4241 | 0.978 | 1.000 |
|  |  |  |  |  |  |  | rs7897619 | 21415 | 0.829 | 0.953 |
|  |  |  |  |  |  |  | rs3780878 | 30494 | 0.829 | 0.953 |
|  |  |  |  |  |  |  | rs11203099 | 66934 | 0.808 | 0.930 |
| rs3771300 | G: 0.481 | 2 | STAT1 | intron |  |  | rs3771300 | 0 | 1.000 | 1.000 |
|  |  |  |  |  |  |  | rs3088307 | 6184 | 0.887 | 1.000 |
|  |  |  |  |  |  |  | rs1607187 | 20558 | 0.855 | 0.982 |
| rs3782415 | C: 0.222 | 12 | SOCS2 | intron |  |  | rs3782415 | 0 | 1.000 | 1.000 |
|  |  |  |  |  |  |  | rs2053196 | 4796 | 1.000 | 1.000 |
|  |  |  |  |  |  |  | rs10777530 | 6645 | 0.950 | 0.975 |
| rs3921 | C: 0.492 | 4 | CXCL10 | 3UTR |  |  | rs3921 | 0 | 1.000 | 1.000 |
| rs462698 | G: 0.411 | 21 | MX1 | intron |  |  | rs462698 | 0 | 1.000 | 1.000 |
|  |  |  |  |  |  |  | rs469288 | 1516 | 1.000 | 1.000 |
|  |  |  |  |  |  |  | rs469390 | 1864 | 1.000 | 1.000 |
|  |  |  |  |  |  |  | rs467773 | 2040 | 1.000 | 1.000 |
|  |  |  |  |  |  |  | rs364199 | 2066 | 1.000 | 1.000 |
|  |  |  |  |  |  |  | rs469483 | 2449 | 1.000 | 1.000 |
|  |  |  |  |  |  |  | rs364197 | 697 | 0.930 | 1.000 |
|  |  |  |  |  |  |  | rs468440 | 1545 | 0.930 | 1.000 |
| rs4780355 | C: 0.334 | 16 | SOCS1 | flanking_3UTR |  |  | rs4780355 | 0 | 1.000 | 1.000 |
|  |  |  |  |  |  |  | rs243323 | 13344 | 0.938 | 0.979 |
|  |  |  |  |  |  |  | rs243325 | 6639 | 0.881 | 0.978 |
| rs4890707 | C: 0.438 | 18 | PIAS2 | flanking_5UTR |  |  | rs4890707 | 0 | 1.000 | 1.000 |
| rs4916014 | G: 0.277 | 1 | JAK1 | flanking_5UTR |  |  | rs4916014 | 0 | 1.000 | 1.000 |
| rs4969168 | A: 0.147 | 17 | SOCS3 | 3UTR |  |  | rs4969168 | 0 | 1.000 | 1.000 |
| rs4969170 | A: 0.353 | 17 | SOCS3 | flanking_5UTR |  |  | rs4969170 | 0 | 1.000 | 1.000 |
|  |  |  |  |  |  |  | rs4969172 | 5253 | 0.963 | 0.981 |
| rs627928 | A: 0.431 | 1 | RNASEL | 182582202 |  |  | rs627928 | 0 | 1.000 | 1.000 |
|  |  |  |  |  |  |  | rs611280 | 1406 | 1.000 | 1.000 |
| rs7135577 | A: 0.345 | 12 | OAS1 | 112920301 |  |  | rs7135577 | 0 | 1.000 | 1.000 |
| rs7138267 | T: 0.259 | 12 | OAS3 | 3UTR |  |  | rs7138267 | 0 | 1.000 | 1.000 |
|  |  |  |  |  |  |  | rs7023 | 994 | 0.958 | 1.000 |
|  |  |  |  |  |  |  | rs739903 | 1107 | 0.958 | 1.000 |
|  |  |  |  |  |  |  | rs757404 | 2789 | 0.958 | 1.000 |
|  |  |  |  |  |  |  | rs2240187 | 5986 | 0.958 | 1.000 |
|  |  |  |  |  |  |  | rs2240188 | 6297 | 0.958 | 1.000 |
|  |  |  |  |  |  |  | rs2240189 | 6484 | 0.958 | 1.000 |
|  |  |  |  |  |  |  | rs2072135 | 10802 | 0.938 | 1.000 |
|  |  |  |  |  |  |  | rs7965570 | 23357 | 0.938 | 1.000 |
|  |  |  |  |  |  |  | rs2384075 | 6672 | 0.860 | 0.957 |
|  |  |  |  |  |  |  | rs12425514 | 15556 | 0.822 | 0.936 |
| rs7257871 | C: 0.236 | 19 | ICAM3 | 10338576 |  |  | rs7257871 | 0 | 1.000 | 1.000 |
|  |  |  |  |  |  |  | rs7258015 | 106 | 1.000 | 1.000 |
|  |  |  |  |  |  |  | rs3176768 | 413 | 1.000 | 1.000 |
|  |  |  |  |  |  |  | rs3176767 | 499 | 1.000 | 1.000 |
|  |  |  |  |  |  |  | rs2304237 | 2684 | 0.932 | 0.977 |
| rs7514391 | G: 0.418 | 1 | IFI44 | intron |  |  | rs7514391 | 0 | 1.000 | 1.000 |
| rs7977692 | G: 0.002 | 12 | STAT2 | flanking_3UTR |  |  | rs7977692 | 0 | 1.000 | 1.000 |
| rs8064821 | A: 0.124 | 17 | SOCS3 | flanking_5UTR |  |  | rs8064821 | 0 | 1.000 | 1.000 |
|  |  |  |  |  |  |  | rs7220865 | 20282 | 0.948 | 1.000 |
|  |  |  |  |  |  |  | rs11871028 | 47264 | 0.898 | 0.948 |
|  |  |  |  |  |  |  | rs8069695 | 27653 | 0.853 | 0.947 |
| rs9283487 | NA | 2 | PRKRA | 3UTR |  |  | rs9283487 | 0 | 1.000 | 1.000 |

NA: not available for CEU population
