# Supplementary material for: Interferon-related genetic markers of necroinflammatory activity in chronic hepatitis C
Source: PLoS One. 2017 Jul 12;12(7):e0180927. doi: 10.1371/journal.pone.0180927 (PMC5507534; doi:10.1371/journal.pone.0180927)
Supplement: S2 Table — (DOCX) [file pone.0180927.s002.docx]

**S2 Table. Genotypic distribution of the analyzed SPNs in the overall population of CHC patients and stratified by necroinflammatory activity grade.**

|  |  |  | All subjects | | NIA ≤ 2 | | NIA = 3 | |
| --- | --- | --- | --- | --- | --- | --- | --- | --- |
| Locus | SNP | Genotype | number | % | number | % | number | % |
| IFNL4 | rs12979860 | C/C | 35 | 0.30 | 20 | 0.25 | 15 | 0.38 |
|  |  | C/T | 66 | 0.56 | 44 | 0.56 | 22 | 0.56 |
|  |  | T/T | 17 | 0.14 | 15 | 0.19 | 2 | 0.05 |
|  |  | NA | 1 | --- | 1 | --- | 0 | --- |
| STAT1 | rs2030171 | A/A | 12 | 0.12 | 7 | 0.11 | 5 | 0.15 |
|  |  | G/A | 41 | 0.42 | 29 | 0.45 | 12 | 0.35 |
|  |  | G/G | 45 | 0.46 | 28 | 0.44 | 17 | 0.50 |
|  |  | NA | 21 | --- | 16 | --- | 5 | --- |
| JAK1 | rs1497056 | A/A | 81 | 0.74 | 60 | 0.81 | 21 | 0.60 |
|  |  | A/G | 26 | 0.24 | 13 | 0.18 | 13 | 0.37 |
|  |  | G/G | 2 | 0.02 | 1 | 0.01 | 1 | 0.03 |
|  |  | NA | 10 | --- | 6 | --- | 4 | --- |
| OAS3 | rs2285933 | C/C | 14 | 0.12 | 11 | 0.14 | 3 | 0.08 |
|  |  | G/C | 45 | 0.39 | 33 | 0.42 | 12 | 0.32 |
|  |  | G/G | 57 | 0.49 | 35 | 0.44 | 22 | 0.59 |
|  |  | NA | 3 | --- | 1 | --- | 2 | --- |
| OAS1 | rs2057778 | A/A | 53 | 0.46 | 36 | 0.47 | 17 | 0.44 |
|  |  | A/C | 55 | 0.48 | 38 | 0.50 | 17 | 0.44 |
|  |  | C/C | 7 | 0.06 | 2 | 0.03 | 5 | 0.13 |
|  |  | NA | 4 | --- | 4 | --- | 0 | --- |
| OAS2 | rs1293748 | A/A | 10 | 0.09 | 5 | 0.07 | 5 | 0.13 |
|  |  | G/A | 52 | 0.46 | 35 | 0.47 | 17 | 0.45 |
|  |  | G/G | 50 | 0.45 | 34 | 0.46 | 16 | 0.42 |
|  |  | NA | 7 | --- | 6 | --- | 1 | --- |
| IFIT2 | rs3740027 | A/A | 11 | 0.11 | 6 | 0.08 | 5 | 0.16 |
|  |  | C/A | 41 | 0.39 | 30 | 0.42 | 11 | 0.34 |
|  |  | C/C | 52 | 0.50 | 36 | 0.50 | 16 | 0.50 |
|  |  | NA | 15 | --- | 8 | --- | 7 | --- |
| BCL2L12 | rs2304206 | G/G | 63 | 0.62 | 41 | 0.6 | 22 | 0.67 |
|  |  | G/A | 38 | 0.38 | 27 | 0.40 | 11 | 0.33 |
|  |  | A/A | 0 | 0 | 0 | 0 | 0 | 0 |
|  |  | NA | 18 | --- | 12 | --- | 6 | --- |
| IFNAR2 | rs3153 | A/A | 7 | 0.07 | 3 | 0.05 | 4 | 0.11 |
|  |  | G/A | 51 | 0.50 | 34 | 0.52 | 17 | 0.47 |
|  |  | G/G | 44 | 0.43 | 29 | 0.44 | 15 | 0.42 |
|  |  | NA | 17 | --- | 14 | --- | 3 | --- |
| JAK1 | rs4916014 | A/A | 63 | 0.57 | 46 | 0.61 | 17 | 0.47 |
|  |  | A/G | 45 | 0.41 | 28 | 0.37 | 17 | 0.47 |
|  |  | G/G | 3 | 0.03 | 1 | 0.01 | 2 | 0.06 |
|  |  | NA | 8 | --- | 5 | --- | 3 | --- |
| IFI44 | rs7514391 | A/A | 46 | 0.41 | 30 | 0.39 | 16 | 0.44 |
|  |  | A/G | 49 | 0.43 | 35 | 0.45 | 14 | 0.39 |
|  |  | G/G | 18 | 0.16 | 12 | 0.16 | 6 | 0.17 |
|  |  | NA | 6 | --- | 3 | --- | 3 | --- |
| OAS1 | rs7135577 | A/A | 9 | 0.08 | 4 | 0.06 | 5 | 0.14 |
|  |  | G/A | 52 | 0.49 | 36 | 0.50 | 16 | 0.46 |
|  |  | G/G | 46 | 0.43 | 32 | 0.44 | 14 | 0.40 |
|  |  | NA | 12 | --- | 8 | --- | 4 | --- |
| JAK1 | rs310209 | A/A | 9 | 0.08 | 6 | 0.08 | 3 | 0.08 |
|  |  | C/A | 40 | 0.34 | 27 | 0.34 | 13 | 0.34 |
|  |  | C/C | 69 | 0.58 | 47 | 0.59 | 22 | 0.58 |
|  |  | NA | 1 | --- | 0 | --- | 1 | --- |
| PIAS2 | rs2032215 | A/A | 24 | 0.24 | 15 | 0.22 | 9 | 0.28 |
|  |  | A/C | 55 | 0.54 | 42 | 0.61 | 13 | 0.41 |
|  |  | C/C | 22 | 0.22 | 12 | 0.17 | 10 | 0.31 |
|  |  | NA | 18 | --- | 11 | --- | 7 | --- |
| PIAS1 | rs1049493 | A/A | 37 | 0.32 | 23 | 0.30 | 14 | 0.38 |
|  |  | A/G | 58 | 0.51 | 40 | 0.52 | 18 | 0.49 |
|  |  | G/G | 19 | 0.17 | 14 | 0.18 | 5 | 0.14 |
|  |  | NA | 5 | --- | 3 | --- | 2 | --- |
| IFIT2 | rs2070845 | A/A | 59 | 0.50 | 41 | 0.52 | 18 | 0.47 |
|  |  | A/G | 47 | 0.40 | 33 | 0.42 | 14 | 0.37 |
|  |  | G/G | 11 | 0.09 | 5 | 0.06 | 6 | 0.16 |
|  |  | NA | 2 | --- | 1 | --- | 1 | --- |
| RNASEL | rs12135247 | A/A | 60 | 0.52 | 37 | 0.48 | 23 | 0.61 |
|  |  | A/G | 40 | 0.35 | 28 | 0.36 | 12 | 0.32 |
|  |  | G/G | 15 | 0.13 | 12 | 0.16 | 3 | 0.08 |
|  |  | NA | 4 | --- | 3 | --- | 1 | --- |
| SOCS3 | rs4969168 | G/G | 77 | 0.67 | 53 | 0.68 | 24 | 0.65 |
|  |  | G/A | 38 | 0.33 | 25 | 0.32 | 13 | 0.35 |
|  |  | A/A | 0 | 0 | 0 | 0 | 0 | 0 |
|  |  | NA | 4 | --- | 2 | --- | 2 | --- |
| RNASEL | rs3738579 | A/A | 42 | 0.37 | 33 | 0.43 | 9 | 0.24 |
|  |  | A/G | 55 | 0.48 | 34 | 0.45 | 21 | 0.55 |
|  |  | G/G | 17 | 0.15 | 9 | 0.12 | 8 | 0.21 |
|  |  | NA | 5 | --- | 4 | --- | 1 | --- |
| KIAA1542 | rs12805435 | A/A | 53 | 0.50 | 36 | 0.51 | 17 | 0.49 |
|  |  | A/G | 48 | 0.45 | 33 | 0.46 | 15 | 0.43 |
|  |  | G/G | 5 | 0.05 | 2 | 0.03 | 3 | 0.09 |
|  |  | NA | 13 | --- | 9 | --- | 4 | --- |
| OASL | rs12819210 | A/A | 2 | 0.02 | 1 | 0.02 | 1 | 0.03 |
|  |  | G/A | 24 | 0.24 | 16 | 0.25 | 8 | 0.22 |
|  |  | G/G | 76 | 0.75 | 48 | 0.74 | 28 | 0.76 |
|  |  | NA | 17 | --- | 15 | --- | 2 | --- |
| PRKRA | rs2059691 | A/A | 15 | 0.14 | 11 | 0.15 | 4 | 0.12 |
|  |  | G/A | 48 | 0.45 | 33 | 0.45 | 15 | 0.45 |
|  |  | G/G | 43 | 0.41 | 29 | 0.4 | 14 | 0.42 |
|  |  | NA | 13 | --- | 7 | --- | 6 | --- |
| IFIT1 | rs304478 | A/A | 30 | 0.27 | 19 | 0.25 | 11 | 0.31 |
|  |  | A/C | 59 | 0.53 | 41 | 0.53 | 18 | 0.51 |
|  |  | C/C | 23 | 0.21 | 17 | 0.22 | 6 | 0.17 |
|  |  | NA | 7 | --- | 3 | --- | 4 | --- |
| OAS2 | rs2384075 | A/A | 9 | 0.08 | 7 | 0.09 | 2 | 0.05 |
|  |  | G/A | 52 | 0.46 | 36 | 0.47 | 16 | 0.43 |
|  |  | G/G | 53 | 0.46 | 34 | 0.44 | 19 | 0.51 |
|  |  | NA | 5 | --- | 3 | --- | 2 | --- |
| SOCS3 | rs4969170 | A/A | 16 | 0.14 | 11 | 0.14 | 5 | 0.13 |
|  |  | G/A | 53 | 0.45 | 37 | 0.47 | 16 | 0.41 |
|  |  | G/G | 49 | 0.42 | 31 | 0.39 | 18 | 0.46 |
|  |  | NA | 1 | --- | 1 | --- | 0 | --- |
| MX1 | rs462698 | A/A | 32 | 0.29 | 24 | 0.32 | 8 | 0.22 |
|  |  | A/G | 57 | 0.51 | 33 | 0.44 | 24 | 0.67 |
|  |  | G/G | 22 | 0.2 | 18 | 0.24 | 4 | 0.11 |
|  |  | NA | 8 | --- | 5 | --- | 3 | --- |
| IFNAR1 | rs2834202 | A/A | 70 | 0.59 | 46 | 0.57 | 24 | 0.63 |
|  |  | A/G | 42 | 0.36 | 30 | 0.38 | 12 | 0.32 |
|  |  | G/G | 6 | 0.05 | 4 | 0.05 | 2 | 0.05 |
|  |  | NA | 1 | --- | 0 | --- | 1 | --- |
| CXCL10 | rs3921 | C/C | 41 | 0.35 | 25 | 0.32 | 16 | 0.42 |
|  |  | C/G | 57 | 0.49 | 39 | 0.49 | 18 | 0.47 |
|  |  | G/G | 19 | 0.16 | 15 | 0.19 | 4 | 0.11 |
|  |  | NA | 2 | --- | 1 | --- | 1 | --- |
| IRF9 | rs12436555 | A/A | 3 | 0.03 | 2 | 0.03 | 1 | 0.03 |
|  |  | G/A | 25 | 0.23 | 20 | 0.26 | 5 | 0.14 |
|  |  | G/G | 83 | 0.75 | 54 | 0.71 | 29 | 0.83 |
|  |  | NA | 8 | --- | 4 | --- | 4 | --- |
| ICAM3 | rs7257871 | A/A | 71 | 0.60 | 46 | 0.57 | 25 | 0.64 |
|  |  | A/G | 44 | 0.37 | 30 | 0.38 | 14 | 0.36 |
|  |  | G/G | 4 | 0.03 | 4 | 0.05 | 0 | 0 |
| IFIT1 | rs303217 | A/A | 22 | 0.19 | 16 | 0.20 | 6 | 0.15 |
|  |  | G/A | 66 | 0.56 | 45 | 0.57 | 21 | 0.54 |
|  |  | G/G | 30 | 0.25 | 18 | 0.23 | 12 | 0.31 |
|  |  | NA | 1 | --- | 1 | --- | 0 | --- |
| PRKRA | rs9283487 | A/A | 67 | 0.61 | 47 | 0.63 | 20 | 0.59 |
|  |  | A/G | 42 | 0.39 | 28 | 0.37 | 14 | 0.41 |
|  |  | G/G | 0 | 0 | 0 | 0 | 0 | 0 |
|  |  | NA | 10 | --- | 5 | --- | 5 | --- |
| APOF | rs11575221 | A/A | 117 | 1 | 80 | 1 | 39 | 1 |
|  |  | NA | 2 | --- | 2 | --- | 0 | --- |
| IFNAR2 | rs17860115 | A/A | 4 | 0.07 | 2 | 0.05 | 2 | 0.14 |
|  |  | C/A | 24 | 0.44 | 17 | 0.42 | 7 | 0.50 |
|  |  | C/C | 26 | 0.48 | 21 | 0.52 | 5 | 0.36 |
|  |  | NA | 65 | --- | 40 | --- | 25 | --- |
| OAS2 | rs1293764 | A/A | 10 | 0.10 | 5 | 0.07 | 5 | 0.16 |
|  |  | G/A | 51 | 0.49 | 39 | 0.53 | 12 | 0.39 |
|  |  | G/G | 43 | 0.41 | 29 | 0.40 | 14 | 0.45 |
|  |  | NA | 15 | --- | 7 | --- | 8 | --- |
| EIF2S1 | rs2031564 | A/A | 7 | 0.06 | 4 | 0.05 | 3 | 0.08 |
|  |  | G/A | 37 | 0.32 | 24 | 0.30 | 13 | 0.36 |
|  |  | G/G | 72 | 0.62 | 52 | 0.65 | 20 | 0.56 |
|  |  | NA | 3 | --- | 0 | --- | 3 | --- |
| SOCS1 | rs4780355 | A/A | 41 | 0.36 | 25 | 0.32 | 16 | 0.42 |
|  |  | A/G | 62 | 0.54 | 44 | 0.57 | 18 | 0.47 |
|  |  | G/G | 12 | 0.1 | 8 | 0.10 | 4 | 0.11 |
|  |  | NA | 4 | --- | 3 | --- | 1 | --- |
| JAK1 | rs11576173 | A/A | 5 | 0.05 | 1 | 0.01 | 4 | 0.11 |
|  |  | G/A | 52 | 0.48 | 34 | 0.48 | 18 | 0.47 |
|  |  | G/G | 52 | 0.48 | 36 | 0.51 | 16 | 0.42 |
|  |  | NA | 10 | --- | 9 | --- | 1 | --- |
| OAS3 | rs10735079 | A/A | 45 | 0.39 | 31 | 0.40 | 14 | 0.37 |
|  |  | A/G | 60 | 0.52 | 41 | 0.53 | 19 | 0.50 |
|  |  | G/G | 11 | 0.09 | 6 | 0.08 | 5 | 0.13 |
|  |  | NA | 3 | --- | 2 | --- | 1 | --- |
| SOCS3 | rs8064821 | A/A | 3 | 0.03 | 3 | 0.04 | 0 | 0 |
|  |  | C/A | 29 | 0.27 | 18 | 0.26 | 11 | 0.30 |
|  |  | C/C | 73 | 0.70 | 47 | 0.69 | 26 | 0.70 |
|  |  | NA | 14 | --- | 12 | --- | 2 | --- |
| PIAS2 | rs10502878 | A/A | 4 | 0.04 | 3 | 0.04 | 1 | 0.03 |
|  |  | G/A | 48 | 0.44 | 34 | 0.46 | 14 | 0.40 |
|  |  | G/G | 57 | 0.52 | 37 | 0.50 | 20 | 0.57 |
|  |  | NA | 10 | --- | 6 | --- | 4 | --- |
| PIAS2 | rs4890707 | C/C | 19 | 0.19 | 11 | 0.16 | 8 | 0.24 |
|  |  | G/C | 51 | 0.50 | 38 | 0.57 | 13 | 0.38 |
|  |  | G/G | 31 | 0.31 | 18 | 0.27 | 13 | 0.38 |
|  |  | NA | 18 | --- | 13 | --- | 5 | --- |
| RNASEL | rs627928 | A/A | 24 | 0.21 | 20 | 0.25 | 4 | 0.11 |
|  |  | C/A | 51 | 0.44 | 34 | 0.43 | 17 | 0.46 |
|  |  | C/C | 41 | 0.35 | 25 | 0.32 | 16 | 0.43 |
|  |  | NA | 3 | --- | 1 | --- | 2 | --- |
| JAK1 | rs310245 | A/A | 14 | 0.12 | 9 | 0.12 | 5 | 0.13 |
|  |  | G/A | 55 | 0.47 | 35 | 0.45 | 20 | 0.53 |
|  |  | G/G | 47 | 0.41 | 34 | 0.44 | 13 | 0.34 |
|  |  | NA | 3 | --- | 2 | --- | 1 | --- |
| SOCS2 | rs2072593 | A/A | 84 | 0.71 | 59 | 0.75 | 25 | 0.64 |
|  |  | A/G | 30 | 0.25 | 18 | 0.23 | 12 | 0.31 |
|  |  | G/G | 4 | 0.03 | 2 | 0.03 | 2 | 0.05 |
|  |  | NA | 1 | --- | 1 | --- | 0 | --- |
| SOCS1 | rs243330 | A/A | 24 | 0.21 | 17 | 0.22 | 7 | 0.18 |
|  |  | G/A | 60 | 0.51 | 38 | 0.49 | 22 | 0.56 |
|  |  | G/G | 33 | 0.28 | 23 | 0.29 | 10 | 0.26 |
|  |  | NA | 2 | --- | 2 | --- | 0 | --- |
| IRF3 | rs10415576 | A/A | 54 | 0.46 | 35 | 0.44 | 19 | 0.49 |
|  |  | A/G | 47 | 0.40 | 30 | 0.38 | 17 | 0.44 |
|  |  | G/G | 17 | 0.14 | 14 | 0.18 | 3 | 0.08 |
|  |  | NA | 1 | --- | 1 | --- | 0 | --- |
| OAS2 | rs1293767 | C/C | 10 | 0.09 | 5 | 0.06 | 5 | 0.13 |
|  |  | G/C | 57 | 0.49 | 41 | 0.53 | 16 | 0.42 |
|  |  | G/G | 49 | 0.42 | 32 | 0.41 | 17 | 0.45 |
|  |  | NA | 3 | --- | 2 | --- | 1 | --- |
| OAS3 | rs12819767 | A/A | 0 | 0 | 0 | 0 | 0 | 0 |
|  |  | G/A | 5 | 0.05 | 3 | 0.04 | 2 | 0.05 |
|  |  | G/G | 104 | 0.95 | 69 | 0.96 | 35 | 0.95 |
|  |  | NA | 10 | --- | 8 | --- | 2 | --- |
| STAT2 | rs7977692 | A/A | 118 | 1 | 80 | 1 | 39 | 1 |
|  |  | NA | 1 | --- | 1 | --- | 0 | --- |
| OAS1 | rs1051042 | C/C | 51 | 0.44 | 35 | 0.45 | 16 | 0.42 |
|  |  | C/G | 55 | 0.48 | 38 | 0.49 | 17 | 0.45 |
|  |  | G/G | 9 | 0.08 | 4 | 0.05 | 5 | 0.13 |
|  |  | NA | 4 | --- | 3 | --- | 1 | --- |
| PRKRA | rs10207436 | A/A | 68 | 0.59 | 45 | 0.57 | 23 | 0.62 |
|  |  | A/G | 42 | 0.36 | 31 | 0.39 | 11 | 0.30 |
|  |  | G/G | 6 | 0.05 | 3 | 0.04 | 3 | 0.08 |
|  |  | NA | 3 | --- | 1 | --- | 2 | --- |
| IFNAR2 | rs17860241 | A/A | 50 | 0.43 | 36 | 0.46 | 14 | 0.37 |
|  |  | A/G | 58 | 0.50 | 38 | 0.48 | 20 | 0.53 |
|  |  | G/G | 9 | 0.08 | 5 | 0.06 | 4 | 0.11 |
|  |  | NA | 2 | --- | 1 | --- | 1 | --- |
| SOCS2 | rs3782415 | A/A | 82 | 0.70 | 57 | 0.72 | 25 | 0.66 |
|  |  | A/G | 31 | 0.26 | 20 | 0.25 | 11 | 0.29 |
|  |  | G/G | 4 | 0.03 | 2 | 0.03 | 2 | 0.05 |
|  |  | NA | 2 | --- | 1 | --- | 1 | --- |
| OAS3 | rs7138267 | A/A | 10 | 0.09 | 8 | 0.10 | 2 | 0.05 |
|  |  | G/A | 49 | 0.43 | 33 | 0.43 | 16 | 0.42 |
|  |  | G/G | 56 | 0.49 | 36 | 0.47 | 20 | 0.53 |
|  |  | NA | 4 | --- | 3 | --- | 1 | --- |
| TYK2 | rs280519 | A/A | 23 | 0.21 | 20 | 0.29 | 3 | 0.08 |
|  |  | G/A | 57 | 0.53 | 34 | 0.49 | 23 | 0.62 |
|  |  | G/G | 27 | 0.25 | 16 | 0.23 | 11 | 0.30 |
|  |  | NA | 12 | --- | 10 | --- | 2 | --- |
| OASL | rs3213545 | A/A | 13 | 0.12 | 10 | 0.14 | 3 | 0.09 |
|  |  | G/A | 36 | 0.33 | 26 | 0.35 | 10 | 0.29 |
|  |  | G/G | 60 | 0.55 | 38 | 0.51 | 22 | 0.63 |
|  |  | NA | 10 | --- | 6 | --- | 4 | --- |
| APOF | rs11575216 | G/G | 117 | 1 | 80 | 1 | 39 | 1 |
|  |  | NA | 2 | --- | 0 | --- | 2 | --- |
| SOCS1 | rs33932899 | C/C | 66 | 0.59 | 47 | 0.63 | 19 | 0.51 |
|  |  | C/G | 43 | 0.38 | 28 | 0.37 | 15 | 0.41 |
|  |  | G/G | 3 | 0.03 | 0 | 0 | 3 | 0.08 |
|  |  | NA | 7 | --- | 5 | --- | 2 | --- |
| RNASEL | rs1048260 | C/C | 62 | 0.52 | 39 | 0.49 | 23 | 0.59 |
|  |  | C/G | 42 | 0.35 | 30 | 0.38 | 12 | 0.31 |
|  |  | G/G | 15 | 0.13 | 11 | 0.14 | 4 | 0.10 |
| STAT1 | rs10208033 | A/A | 36 | 0.31 | 23 | 0.30 | 13 | 0.33 |
|  |  | A/G | 62 | 0.53 | 42 | 0.55 | 20 | 0.51 |
|  |  | G/G | 18 | 0.16 | 12 | 0.16 | 6 | 0.15 |
|  |  | NA | 3 | --- | 3 | --- | 0 | --- |
| JAK1 | rs310216 | A/A | 8 | 0.07 | 6 | 0.08 | 2 | 0.06 |
|  |  | G/A | 35 | 0.32 | 23 | 0.31 | 12 | 0.34 |
|  |  | G/G | 66 | 0.61 | 45 | 0.61 | 21 | 0.60 |
|  |  | NA | 10 | --- | 6 | --- | 4 | --- |
| STAT1 | rs3771300 | A/A | 31 | 0.27 | 21 | 0.27 | 10 | 0.27 |
|  |  | A/C | 60 | 0.53 | 44 | 0.57 | 16 | 0.43 |
|  |  | C/C | 23 | 0.20 | 12 | 0.16 | 11 | 0.30 |
|  |  | NA | 5 | --- | 3 | --- | 2 | --- |
