# Supplementary material for: Interferon-related genetic markers of necroinflammatory activity in chronic hepatitis C
Source: PLoS One. 2017 Jul 12;12(7):e0180927. doi: 10.1371/journal.pone.0180927 (PMC5507534; doi:10.1371/journal.pone.0180927)
Supplement: S3 Table — (DOCX) [file pone.0180927.s003.docx]

**S3 Table. Distribution of main clinical variables among *TYK2* rs280519 and *RNASEL* rs3738579 genotypes.**

| SNP | Genotype | VLx10^-5^ (IU/mL) | | AST (IU/L) | | ALT (IU/L) | | GGT (IU/L) | | Fibrosis [N (%)] | | |
| --- | --- | --- | --- | --- | --- | --- | --- | --- | --- | --- | --- | --- |
|  |  | [mean (SD)] | p | [mean (SD)] | p | [mean (SD)] | p | [mean (SD)] | p | F ≤ 2 | F > 2 | p |
| rs3738579 | A/A | 22.40 (43.06) | 0.95 | 59.52 (31.25) | 0.66 | 99.95 (66.16) | 0.55 | 74.93 (65.8) | 0.33 | 28 (39%) | 5 (28%) | 0.61 |
|  | A/G | 16.70 (18.02) |  | 57.66 (35.77) |  | 86.40 (51.24) |  | 54.42 (45.43) |  | 31 (43%) | 10 (56%) |  |
|  | G/G | 17.38 (15.98) |  | 63.88 (48.09) |  | 110.53 (76.82) |  | 64.18 (50.36) |  | 13 (18%) | 3 (17%) |  |
| rs280519 | A/A | 24.95 (53.57) | 0.08 | 65.23 (44.98) | 0.91 | 105.36 (78.1) | 0.97 | 88.55 (76.58) | 0.19 | 14 (21%) | 3 (19%) | 0.90 |
|  | A/G | 19.32 (22.59) |  | 60.18 (37.03) |  | 98.27 (62.13) |  | 60.80 (47.04) |  | 35 (53%) | 8 (50%) |  |
|  | G/G | 11.29 (12.71) |  | 59.78 (29.57) |  | 93.00 (45.8) |  | 57.96 (49.31) |  | 17 (26%) | 5 (31%) |  |

None of the analyzed variables showed a significant relation with the SNPs (Kruskal-Walllis test for continuous variables and Chi-squared test for Fibrosis, p>0.05).

VL, viral load; IU, international units; AST, aspartate aminotransferase; ALT, alanine transaminase; GGT, gamma glutamyl transferase.
