# Supplementary material for: Interferon-related genetic markers of necroinflammatory activity in chronic hepatitis C
Source: PLoS One. 2017 Jul 12;12(7):e0180927. doi: 10.1371/journal.pone.0180927 (PMC5507534; doi:10.1371/journal.pone.0180927)
Supplement: S4 Table — (DOCX) [file pone.0180927.s004.docx]

**S4 Table.** [**Variants linked to *TYK2* rs280519 and *RNASEL* rs3738579**](http://www.ensembl.org/Homo_sapiens/Variation/HighLD?db=core;r=19:10361757-10362757;v=rs280519;vdb=variation;vf=162941#373513_table)**.**

| SNP | SNP in LD | Location | Distance (bp) | r^2^ | D' | Consequence Type | Located in gene | Allele | VEP [36] |
| --- | --- | --- | --- | --- | --- | --- | --- | --- | --- |
| rs280519 | rs91755 | 19:10362894 | 637 | 1 | 1 | Intron variant | TYK2 | G | intron_variant |
|  | rs280525 | 19:10370835 | 8578 | 0.96 | 1 | Intron variant | TYK2 | A | intron_variant |
|  | rs280526 | 19:10370856 | 8599 | 0.96 | 1 | Intron variant | TYK2 | G | intron_variant |
|  | rs280497 | 19:10354011 | 8246 | 0.94 | 0.979 | Non coding transcript exon variant | TYK2 | G | TF_binding_site_variant |
| rs3738579 | rs486907 | 1:182585422 | 1479 | 0.978 | 1 | Missense variant | RNASEL | T | missense_variant |
|  | rs579006 | 1:182580201 | 6700 | 0.978 | 1 | Intron variant | RNASEL | G | intron_variant |
|  | rs12041279 | 1:182590629 | 3728 | 0.913 | 0.977 | Upstream gene variant | - | A | upstream_gene_variant |
|  | rs12042348 | 1:182592128 | 5227 | 0.913 | 0.977 | Upstream gene variant | - | A | upstream_gene_variant |
|  | rs12041623 | 1:182592498 | 5597 | 0.913 | 0.977 | Upstream gene variant | - | C | upstream_gene_variant |
|  | rs34335333 | 1:182592917 | 6016 | 0.913 | 0.977 | Upstream gene variant | - | C | upstream_gene_variant |
|  | rs4652733 | 1:182598305 | 11404 | 0.913 | 0.977 | Downstream gene variant | - | A | downstream_gene_variant |
|  | rs3795485 | 1:182598604 | 11703 | 0.913 | 0.977 | Downstream gene variant | - | G | downstream_gene_variant |
|  | rs10489966 | 1:182606241 | 19340 | 0.913 | 0.977 | Upstream gene variant | - | G | upstream_gene_variant |
|  | rs3754289 | 1:182606536 | 19635 | 0.913 | 0.977 | Upstream gene variant | - | A | upstream_gene_variant |

SNP, Single Nucleotide polymorphism; LD, linkage disequilibrium; bp, base pair; VEP, variant effect predictor (McLaren W, et al. The Ensembl Variant Effect Predictor. Genome Biol. 2016;17: 122)
