# Supplementary figures and images for: Interferon-related genetic markers of necroinflammatory activity in chronic hepatitis C
Source: PLoS One. 2017 Jul 12;12(7):e0180927. doi: 10.1371/journal.pone.0180927 (PMC5507534; doi:10.1371/journal.pone.0180927)

## rs280519 ( CEU )

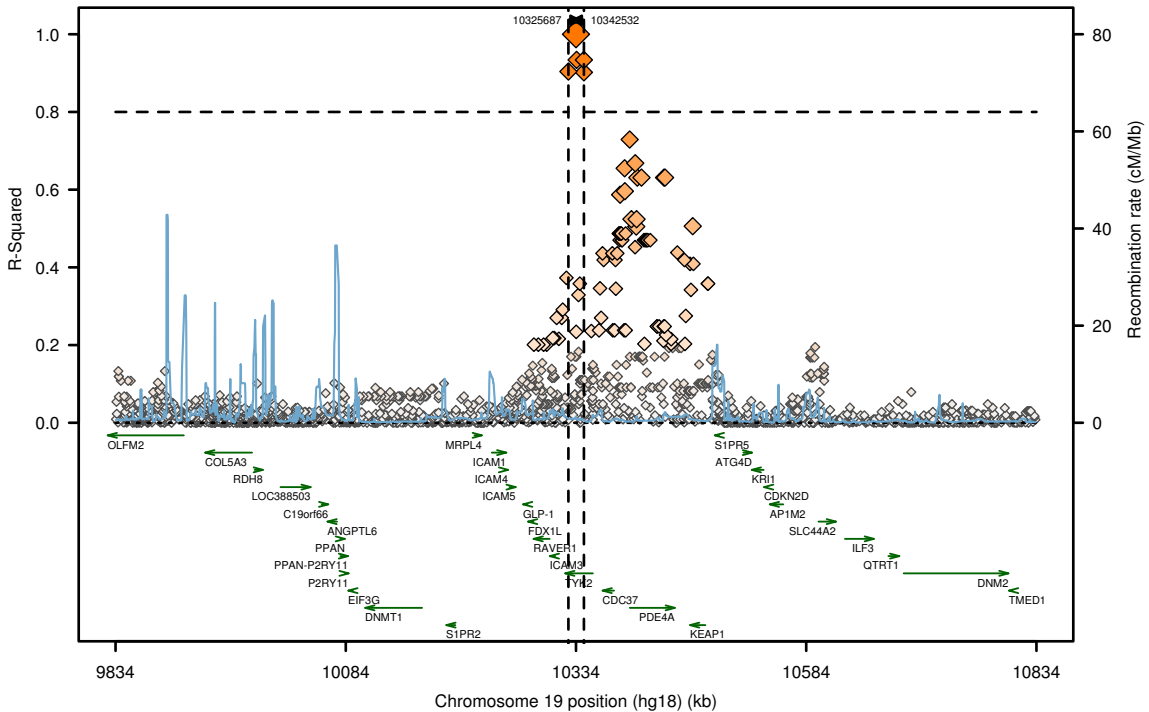

Supplement: S1 Fig — Linkage disequilibrium plot of rs280519 was generated by using the SNP Annotation and Proxy Search tool (SNAP, version 2.2, Broad Institute [38]). Plot was generated under the following conditions: 1000 Genomes Pilot 1 dataset, CEU population panel, r2 threshold = 0.8 and distance limit 500kb. (PDF) [file pone.0180927.s005.pdf]
